# Managing COVID-19 in resource-limited settings: critical care considerations

**DOI:** 10.1186/s13054-020-02890-x

**Published:** 2020-04-22

**Authors:** Wen Ting Siow, Mei Fong Liew, Babu Raja Shrestha, Faisal Muchtar, Kay Choong See

**Affiliations:** 1 Division of Respiratory and Critical Care Medicine, University Medicine Cluster, National University Health System, 1E Kent Ridge Rd, Singapore, 119228 Singapore; 2 Fast and Chronic Programmes, Alexandra Hospital, National University Health System, Queenstown, Singapore; 3 Department of Anaesthesia, Kathmandu Medical College and Teaching Hospital, Kathmandu, Nepal; 4 Department of Anesthesiology, Intensive Care and Pain Management, Medical Faculty, Hasanuddin University, Makassar, South Sulawesi Indonesia

The 2019 coronavirus (COVID-19) pandemic has now involved numerous low-to-middle-income countries (LMICs). The healthcare systems in LMICs face serious constraints in capacity and accessibility during normal times. This would be aggravated during an outbreak, leading to worse clinical outcomes. Moreover, 69% of the global population aged 60 and above live in LMICs. These older persons are at increased risk of severe COVID-19 and mortality [1].

LMICs lack time and finances for swift uptake of new technologies (e.g., rapid test kits, vaccines, and antivirals). From a more urgent and pragmatic perspective, we believe creative use of existing resources and repurposing others for human medical care are needed (Table 1). We acknowledge that our suggestions may be perceived as controversial, and we wish to emphasize that maximization of conventional healthcare assets should always be done before turning to unconventional solutions.

**Table 1:** Managing COVID-19 when resources are limited

Resource limitation	Specific challenges	Optimal use of existing resources	Repurposing other resources for human medical care
**Infrastructure**	Limited number of isolation beds (negative pressure and normal pressure) for suspected and confirmed COVID-19 patients	• Central monitoring of bed numbers for better visibility and allocation • Inclusion of private hospitals and military hospitals in total bed count • Transforming clinics into inpatient care units • Home as hospital concept with HCW monitoring less ill patients in the community using telemedicine • Mounting fever tents outside emergency departments to better triage and segregate symptomatic patients • Utilize military hospital assets (land-based units; hospital ships) • Use diesel-based electrical generators to cope with energy demands • Early engagement of community leaders • Isolating communities instead of individuals in case of local outbreaks	• Opening field hospitals by converting public facilities (e.g., sports facilities, stadiums, soccer fields) and building open tents to house non-critically ill patients and those who cannot stay at home. Use of industrial fans in these spaces to ensure good ventilation • Tap on portable power and solar generators for electricity to run medical equipment • Conversion of public and commercial facilities (e.g., hotel rooms, chalets, hostels) into quarantine facilities for well patients • Mobilizing the community and restaurants to help prepare and deliver food for HCWs and patients in quarantine facilities • Use of industrial exhaust fans to convert single rooms with normal pressure to negative pressure rooms for isolation in hospitals, especially for ICU • Conversion of veterinary hospitals and deploying medical personnel to accept non-critically ill patients • Cohort all confirmed cases in well-ventilated open cubicles to free-up isolation beds for suspected cases
**Monitoring/testing**	Limited number of accredited test labs/sites, especially in suburbs and regional hospitals Lack of point-of-care-certified test kits at the frontlines and community Lack of sufficient mobile test sites/clinics	• International health organizations should coordinate rapid technology transfer to LMICs. Allowance and early acceptance of rapid test kits • Provide 1 low-cost thermometer per family unit for self-monitoring of temperature • Rely on clinical parameters and examination rather than blood tests to preserve lab capacity (e.g., capillary refill time instead of lactate, qSOFA score to predict deterioration) • Noninvasive manual methods, e.g., manual BP rather than IA lines; SpO2 rather than ABG • Point-of-care ultrasound rather than X-rays/CT scans	• Usage of veterinary facilities including animal devices used for patient monitoring and animal ultrasound devices • Mobilize military forces, community partners, schools, and volunteers to help establish mobile test sites for symptomatic patients. These patients can be issued a stay-home notice after the test. Establish a call-center to rapidly inform patients of results and follow-up action (e.g., contact-tracing)
**Treatment**	Insufficient ICU ventilators Insufficient oxygen supply Insufficient medications	• Use transport ventilators and anesthesia units • Splitting ventilators (i.e., attaching up to 4 COVID-19 patients to the same ventilator), using pressure cycling rather than volume cycling, and with continuous mandatory ventilation • Improvised CPAP (iCPAP) to replace invasive ventilation • Using bag-valve-ETT with PEEP valves • Use portable oxygen concentrators rather than tanks, especially in field hospitals • Early use of prone positioning if oxygenation needs exceed available inspired oxygen supply, even in patients who are not on invasive mechanical ventilation • Enteral hydration, vasopressors (e.g., NG midodrine), antimicrobials rather than using intravenous formulations • Avoid expending resources on experimental therapies	• Non-medical factories or production lines to manufacture medical equipment like face masks, ventilators, monitoring devices, and intravenous fluids • Usage of suitable veterinary equipment, e.g., ventilators, IV pumps, and approved drugs, e.g., analgesics, antibiotics, and consumables for wound care
**Personal protective equipment**	Insufficient PPE	• Re-use surgical masks and goggles • Sharing of certain types of PPE like googles after disinfection • Ultraviolet light decontamination of medical equipment, re-used surgical masks and goggles • Use washable gowns and gloves • Use alcohol-based rubs and spirits rather than clean water, which may be in short supply • Assemble reusable elastometric respirators to replace N95 respirators	• Use protective face masks, respirators, and gowns from other industries, e.g., food industries, manufacturing plants, construction, and mining • Getting factories and production lines to manufacture PPE
**Personnel**	Insufficient staff	• Enrolling of dentists, paramedical personnel, village health attendants • Enrolling of military medical personnel • Enrolling of medical, nursing, and allied health students to help with pandemic medical treatment • Designate convalescent HCW to provide care for confirmed COVID-19 patients • Enroll convalescent patients to volunteer at as health attendants	• Enrolling veterinary HCWs and medical students by providing them with crash courses to help stem manpower shortages in hospitals • Enrolling non-medical personnel to act as health attendants, e.g., to do temperature taking and man screening stations. This will relieve workload of existing healthcare personnel
**Information**	Uncertainty and confusion over testing, triage, and treatment	• Setting up protocols and checklists to standardized medical care that are simple, easy to teach. Avoid overuse of non-EBM methods • Promotion of simplified EBM scores for risk stratification, e.g., qSOFA for LMICs • Encourage uptake of teleconferencing platforms to discuss and learn about new updates from international medicine communities	• Use mobile/SMS technology to provide simple policy and health updates to HCWs/public, besides emails and paper-based mailers
**Transport**	Insufficient transport options for patients	• Inclusion of public, commercial, and military healthcare transport vehicles	• Getting nonmedical transport services to become ambulances

*CT* computed tomography, *ETT* endotracheal tube, *HCW* healthcare worker, *LMIC* low-to-middle-income country, *PEEP* positive end expiratory pressure, *PPE* personal protective equipment

## Infrastructure

Anticipation of an impending outbreak helps vital preparation. Unfortunately, during the COVID-19 pandemic, there is little time to construct new infrastructure. The World Health Organization (WHO) has recommended airborne isolation, but isolation facilities are limited. Industrial exhaust fans have been used to convert existing normal pressure single rooms to negative pressure rooms to increase isolation [2]. This approach is relatively quick and may be used for creating more negative pressure intensive care unit (ICU) beds. Alternatively, confirmed cases can be cohorted in open ICUs with adequate ventilation.

Opening field hospitals in large public spaces (e.g., stadiums) allows for triaging and managing stable patients and decongest other hospitals. Local networks between smaller district hospitals and larger tertiary centers can be established to facilitate patient transfers, as smaller hospitals can be easily overwhelmed. Well patients with COVID-19, instead of being quarantined in hospitals, can be quarantined in specially designated facilities, such as a hotel. If communication systems are available, well patients could be sent home and monitored remotely.

Makeshift acute or critical care units can be set up in operating theaters and clinic spaces to cope with increasing numbers of critically ill patients. This can be achieved by reducing non-essential services such as elective surgeries and outpatient clinics.

## Patient monitoring or testing

For LMICs, focused testing on symptomatic patients instead of random screening would place less strain on the healthcare system. Rapid test kits are an option to allow LMICs to perform diagnostic tests faster, but this would require international health organizations to transfer knowledge and test kits. It may be necessary to isolate an entire community for containment.

Radiological investigations and laboratory support would also be stretched beyond capacity. Physicians may need to rely mainly on bedside clinical examination. If available, bedside point-of-care ultrasonography can yield significant amounts of information. Early clinical detection and admission to the correct facility can help with right-siting before confirmatory tests are out, reducing nosocomial and community spread. Simple scoring systems, such as the qSOFA score, can be harnessed to detect deteriorating patients [3].

## Treatment

Hospital ventilators will likely be in shortfall. To supplement ventilators, anesthesia units in operating theaters and transport ventilators can be used. Improvised continuous airway pressure (iCPAP) ventilation systems [4] or bubble continuous airway pressure for children [5] can be employed when there is a dire shortage. The addition of high-efficiency particulate air (HEPA) filters to the expiratory limb of the circuit can help minimize aerosolization if the ventilator does not have a closed circuit. Other creative approaches include splitting a ventilator to support several patients simultaneously by using T-tubes and pressure-cycled ventilation [6–8].

Proning positioning has been reported to work in critically ill COVID-19 patients with moderate-to-severe acute respiratory distress syndrome [1]. Patients who are moderately hypoxemic can be proned early to improve oxygenation if oxygen supplies are limited, presumably even if they are not invasively ventilated [9]. Other approaches to save ICU resources include using enteral vasopressors, such as midodrine for hypotensive patients, instead of intravenous formulations [10]. In states of emergencies, veterinary supplies, such as cleaning solutions, syringe pumps, and even ventilators, could be mobilized to augment hospital stocks.

Ultimately, there still needs to be fair and ethical resource stewardship [11].

## Personal protective equipment (PPE)

Healthcare workers (HCWs) working on the frontlines need to be protected adequately, or they risk catching COVID-19 and even dying [12]. This is also applicable to personnel such as ambulance drivers and military troops. PPE can be reused to reduce waste [13] and preserve existing stocks. Certain types of PPE like goggles may be shared after disinfection. Deploying reusable powered air-purifying respirators is an option. Other innovative approaches include testing, validating, and assembling simple reusable elastometric respirators to replace N95 respirators [14].

If prior infection can be proven to confer immunity, other approaches to reduce PPE use can include deploying convalescent HCWs to care for confirmed COVID-19 patients and enrolling convalescent patients to volunteer in healthcare.

## Personnel

To cope with healthcare demands, active recruitment and training of healthcare personnel should be done concurrently. Besides recalling HCWs on leave, recruitment and redeployment from different sectors (e.g., dentists, paramedics, medical students, military personnel) and even recalling retired personnel may be required. Laypeople, including carers, can be recruited into the hospital after training to provide basic care for patients. Interprofessional skill training should also be done among nurses and doctors. For example, surgeons can be taught simple ventilator care. Sharing simple treatment protocols will aid those who have been redeployed.

## Information

In a large-scale pandemic, crucial information about the disease and workflows are constantly evolving. There is a need for information to be disseminated and assimilated rapidly on the ground to prevent delays. An agile system of information dissemination should include mobile phones via text messages or emails and paper-based mailers. Protocols and checklists will also help in standardizing medical care and reducing wastage.

## Transport

Harnessing non-medical transport services, such as private-hire and public vehicles and military vehicles, can help improve accessibility to healthcare in LMICs. These vehicles can help ferry unwell patients from the suburbs to regional or central hospitals and reduce delays to medical care. A caveat would be that these transport vehicles need to be thoroughly cleaned following transport [15].

## Data Availability

Data sharing is not applicable to this article as no datasets were generated or analyzed during the current study.
